# Cattle Grazing and Conservation of a Meadow-Dependent Amphibian Species in the Sierra Nevada

**DOI:** 10.1371/journal.pone.0035734

**Published:** 2012-04-25

**Authors:** Leslie M. Roche, Andrew M. Latimer, Danny J. Eastburn, Kenneth W. Tate

**Affiliations:** Department of Plant Sciences, University of California Davis, Davis, California, United States of America; State Natural History Museum, Germany

## Abstract

World-wide population declines have sharpened concern for amphibian conservation on working landscapes. Across the Sierra Nevada's national forest lands, where almost half of native amphibian species are considered at risk, permitted livestock grazing is a notably controversial agricultural activity. Cattle (*Bos taurus*) grazing is thought to degrade the quality, and thus reduce occupancy, of meadow breeding habitat for amphibian species of concern such as the endemic Yosemite toad (*Anaxyrus* [ = *Bufo*] *canorus*). However, there is currently little quantitative information correlating cattle grazing intensity, meadow breeding habitat quality, and toad use of meadow habitat. We surveyed biotic and abiotic factors influencing cattle utilization and toad occupancy across 24 Sierra Nevada meadows to establish these correlations and inform conservation planning efforts. We utilized both traditional regression models and Bayesian structural equation modeling to investigate potential drivers of meadow habitat use by cattle and Yosemite toads. Cattle use was negatively related to meadow wetness, while toad occupancy was positively related. In mid and late season (mid July–mid September) grazing periods, cattle selected for higher forage quality diets associated with vegetation in relatively drier meadows, whereas toads were more prevalent in wetter meadows. Because cattle and toads largely occupied divergent zones along the moisture gradient, the potential for indirect or direct negative effects is likely minimized via a partitioning of the meadow habitat. During the early season, when habitat use overlap was highest, overall low grazing levels resulted in no detectable impacts on toad occupancy. Bayesian structural equation analyses supported the hypothesis that meadow hydrology influenced toad meadow occupancy, while cattle grazing intensity did not. These findings suggest cattle production and amphibian conservation can be compatible goals within this working landscape.

## Introduction

Amphibian conservation is gaining considerable attention as a result of increasing quantitative evidence of global population declines [1], [2]. In the Sierra Nevada, nearly half of the native amphibian species are considered at risk by state and federal regulatory agencies [3], [4], . Exotic species introductions, infectious diseases, climate change, and anthropogenic land-use changes such as urbanization and agriculture have all been identified as potential drivers of amphibian declines [7]. Cattle grazing (*Bos taurus*), a prominent agricultural activity in the Sierra Nevada region, has received growing interest as a potential driver [8], [9], 10,11,12,13,14,15, and has been specifically implicated in amphibian species declines in the Sierra Nevada [4], [5], [6], [16].

One of the principal amphibian species of concern for the Sierra Nevada is the Yosemite toad (*Anaxyrus* [ = *Bufo*] *canorus*). Yosemite toad is an amphibian endemic to the Sierra Nevada, and is believed to have disappeared from approximately 50% of its known historic range [4], [17], [18]. Currently, Yosemite toad is a California Species of Special Concern, a U.S. Forest Service Sensitive Species (i.e., species that have exhibited downward trends in population numbers or in habitat capability, thus creating population viability concerns [19]), and a candidate species for federal listing pursuant to the Endangered Species Act [5], [6]. Yosemite toads are typically associated with upper montane and subalpine meadows (ca. 1,950 m to 3,450 m) in the central and southern Sierra Nevada [20], [21]. These mountain meadow habitats exhibit a range of hydrologic conditions varying from scattered, ephemeral pools to expansive, season-long flooded areas, which differentially support toad breeding and rearing habitats.

**Figure 1:**
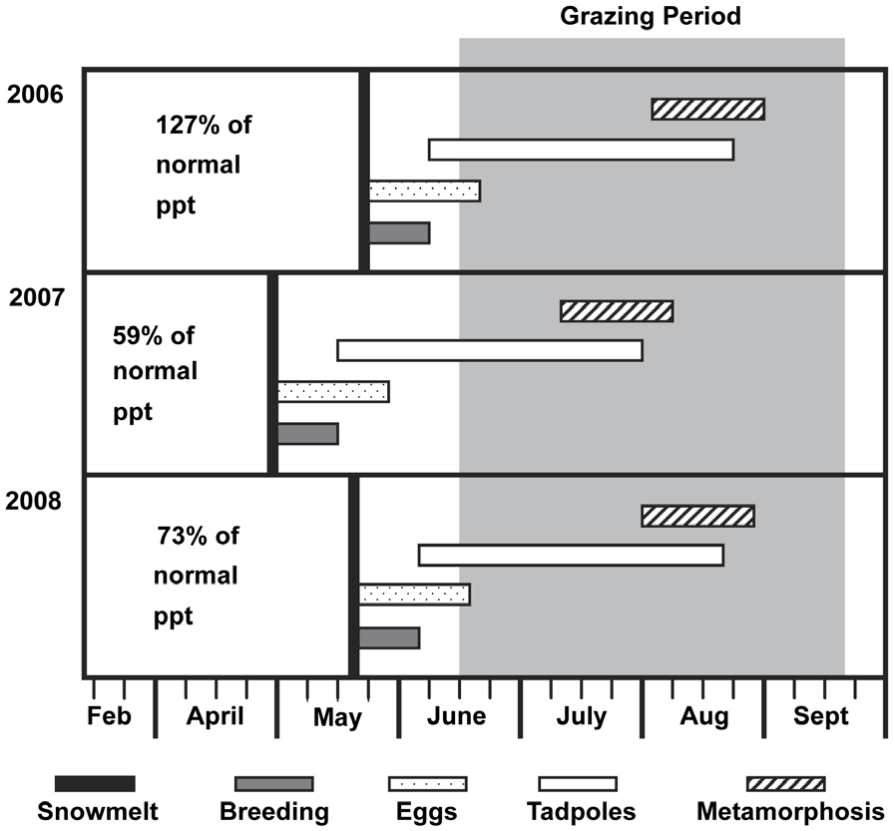
Life stage progression. Diagram illustrating timing of Yosemite toad (*Anaxyrus* [ = *Bufo*] *canorus* Camp) life stages and cattle grazing seasons in the High Sierra Ranger District, Sierra National Forest, California, USA. Data were collected for 2006 to 2008 on cattle grazed meadows in the study area [77], [78].

In addition to supplying vital wildlife habitat distinct from the surrounding forest matrix, mountain meadows also support a critical forage base for permitted cattle grazing in an otherwise depauperate zone [22], [23]. Permitted cattle grazing on the nation's public lands is a notably controversial activity, especially in high-elevation ecosystems. Public lands grazing permits often support low-intensity cattle operations on privately owned foothill ranches. Many Sierran ranching operations depend on these high-elevation rangelands during summer months, when low-elevation grasslands enter the inadequate dry forage period. During this inadequate period, low-elevation forage nutritive quality is generally poor and so managers must seek alternative feed sources (e.g., nutrient supplements, irrigated pastures, high-elevation pastures) to sustain livestock performance and the ranch enterprise [24]. Some suggest broad-scale reductions in public grazing permits would greatly impact the viability of these foothill ranches, forcing ranchers to sell land to developers, which has potentially negative regional socio-economic and ecological implications [25], [26]. However, opponents of public land grazing assert that cattle grazing has intolerable negative impacts on native wildlife and their habitat [27], [28].

In response to growing public concern surrounding cattle-amphibian interactions, some Sierra Nevada grazing permits have been terminated, and seasonal restrictions have been applied to many active permits with known populations of listed sensitive species. These types of management changes, directed to conserve species of concern, are often made despite considerable uncertainty about the system or with key quantitative information lacking. For example, within Sierra Nevada meadow systems, the extent to which cattle and amphibians, such as Yosemite toad, overlap in their habitat needs and use have not yet been jointly addressed. This is a critical knowledge gap because cattle grazing is more likely to have adverse effects if cattle tend to use similar sites as the species of concern, and less likely if they do not. Previous research on cattle-amphibian interactions is largely restricted to ungrazed and grazed (i.e., usually intensively or heavily grazed) comparisons [9], [13], [14], [29], which has limited relevance to systems experiencing extensive grazing (i.e., lower cattle intensities, largely unimproved native pasture systems), such as Sierra Nevada grazing allotments. Additionally, few analyses have applied a systems approach to examining these complex livestock-amphibian interactions at a management scale.

We surveyed meadow characteristics, cattle utilization, and Yosemite toad habitation across a set of Sierra Nevada meadows to simultaneously examine two potential drivers of meadow occupancy by toads: 1) cattle grazing intensity; and 2) meadow wetness (i.e., toad habitat quality). We utilized both traditional bivariate analyses and Bayesian structural equation modeling (SEM) [30], [31] to examine these proposed drivers of meadow occupancy by toads, in addition to potential meadow biotic and abiotic drivers of cattle utilization. SEM has become an effective tool for researchers working in inherently complex natural landscapes, providing greater systems level understanding than traditional approaches [32], [33], [34]. In this analysis, we explicitly asked: 1) how does meadow wetness influence forage quality and herbaceous biomass productivity? 2) what are the relationships between forage quality, forage productivity, and meadow utilization by cattle? and 3) what is the magnitude of influence of current cattle utilization versus meadow wetness on meadow occupancy by Yosemite toads? To address issues of the timing of grazing (i.e., with respect to the toad's lifecycle) that has greatest potential impact, these questions were examined within the seasonal periods in which grazing occurred: early, mid, and late season grazing periods (approximately mid-June through mid-July, mid-July through mid-August, and mid-August through mid-September, respectively).

**Figure 2:**
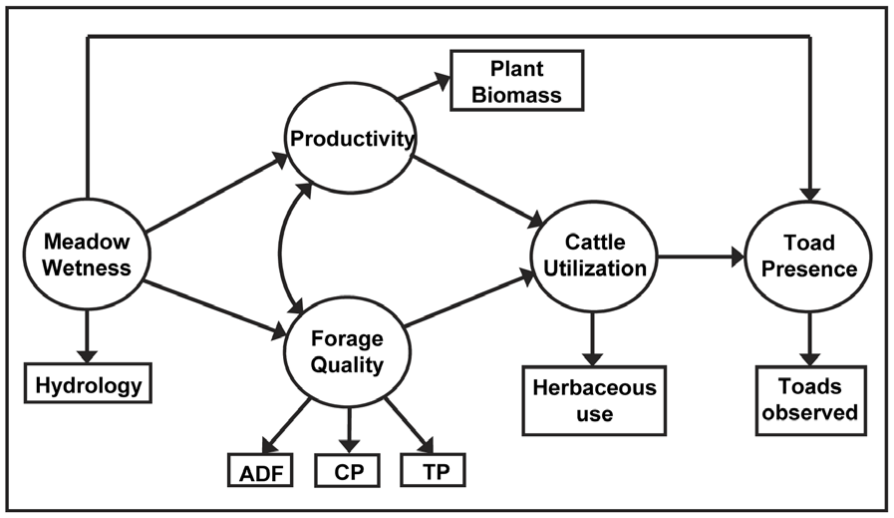
Conceptual Model. Conceptual model of the multiple hypothesized factors influencing toad meadow occupancy in the High Sierra Ranger District, Sierra National Forest, California, USA. Ovals indicate latent variables, which are estimated by observable indicators, represented by boxes. Straight arrows represent direct effects of one variable on another and curved arrows represent correlations between variables.

## Methods

### Ethics statement

This observational field study was conducted in collaboration with the US Forest Service, and so all permissions for site access were granted and no permits were required. We employed sanitary protocols to reduce potential risks of spreading biological contaminants (e.g., the amphibian chytrid *Batrachochytrium dendrobatidis*) between meadows and watersheds. Prior to and following each meadow survey, crew members disinfected their rubber boots with a diluted bleach solution (4% sodium hypochlorite), which has been shown to cause 100% *B. dendrobatidis* mortality with as little as 30 seconds of exposure time [35].

### Study area

This study was conducted on the High Sierra Ranger District of the Sierra National Forest, which is located on the western slope of the central Sierra Nevada in the upper montane zone (2 200 m to 2 700 m). The landscape is a mosaic of meadows, rock outcrops, and coniferous forest dominated by *Pinus contorta*, *Pinus jeffreyi*, *Abies concolor*, and *Abies magnifica*. Meadows, which cover less than 10% of the landscape, are generally characterized by shallow water tables (i.e., near-surface saturated conditions) and accumulations of mineral and organic materials. Within U.S. Forest Service (USFS) managed grazing allotments, 24 meadows providing potential toad breeding and rearing habitat were selected for study. Meadows spanned in elevation from 2 100 m to 2 700 m in elevation, and 0.3 ha to 7.9 ha in size. All meadows were open to cattle grazing under ambient USFS allotment scale management. Allotments ranged from 22 000 to 27 000 hectares with 200 to 250 permitted cow-calf pairs per allotment between mid-June and mid-September (Fig. 1). Soils were classified as Mollisols and Inceptisols with Histosols found in the most saturated zones of meadows [36]. Meadow vegetation was characterized by a dense cover of graminoid and herbaceous species. Meadows with near-surface saturated conditions throughout the growing season were generally dominated by sedges such as *Carex utriculata*, *Carex vesicaria*, and *Carex simulata*. In contrast, meadows experiencing seasonal water table drawdown below the rooting zone were generally dominated by grasses and forbs such as *Deschampsia caespitosa* and *Trifolium* species [36].

Mean annual precipitation in the region is 115 cm, with 70% to 90% falling as snow from October through April. The growing season is relatively short— the region spends approximately 200 days under snowpack annually, with snowmelt typically occurring between May and June. Depending on snowpack depth and timing of melt, Yosemite toads typically emerge from hibernation in late spring (May to June) to breed and lay eggs in shallow pools and flooded areas of meadows. Larvae metamorphose by mid to late summer, and toad metamorphs remain within the breeding and rearing zone for the duration of the summer season [20], [21] (Fig. 1).

### Study design and data collection

We conducted a cross-sectional, longitudinal survey of Yosemite toad occupancy, cattle utilization, vegetation attributes and meadow wetness across 24 meadows over three years (2006 to 2008) on the Sierra National Forest. For purposes of this study, toad occupancy was defined as evidence of breeding (i.e., presence of egg masses, tadpoles, and/or recent metamorphs). Yosemite toad and habitat survey records (conducted in 2002 and 2003; 83% and 94% of mean annual precipitation, respectively) from Forest staff were utilized to define an initial set of meadows with potential to support Yosemite toad breeding populations. From this initial set, we randomly sub-sampled 24 meadows across three grazing allotments. In 2006, five monitoring sites (120 total sites) were established in a stratified random approach across each meadow catena (i.e., a toposequence reflecting effects of topography on proximity to water table and on water movement), representing the major plant communities and moisture gradient in each meadow. Paired 1 m^2^ plots (one cattle grazed plot and one ungrazed caged plot) were randomly located within each plant community/moisture gradient monitoring site, with the ungrazed caged plots relocated within that same site in the second and third years [37], [38].

Cattle utilization and vegetation attributes were recorded at each monitoring site. Cattle utilization was measured via herbaceous utilization (2006 to 2008), which was determined by comparative yield-paired plot methods (Interagency 1996) at the end of the early (July), mid (August), and late (September) season grazing periods each year. In the final year of study (2008), fecal density was measured via three 35 m^2^ belt transects across each meadow to correlate annual utilization levels with a cumulative indicator of recent historic use (5 to 10 yrs). As a result of slow decomposition rates in high-elevation mountain systems, fecal density in mountain meadows represents approximately 5 to 10 years of pat accumulation.

Herbaceous biomass production data (2006 to 2008) and forage samples (2007 and 2008) were collected for each monitoring site in June, July, and August, representing variation in forage characteristics during early, mid, and late seasons, respectively. Herbaceous biomass production was determined via the comparative yield method at ungrazed caged plots [37]. For forage quality analyses, a minimum of 30 grams dry weight was sampled around each paired plot, representing the local plant community patch. Samples were oven-dried at 55°C to 60°C for a minimum of 48 hours, and ground to pass through a 40-mesh screen. Crude protein (CP), acid detergent fiber (ADF), and total phosphorous (TP) were determined for each sample by the University of California Agriculture and Natural Resources Analytical Laboratory, UC Davis, California. CP was directly calculated from sample nitrogen content, which was measured via nitrogen gas analyzer utilizing induction furnace and thermal conductivity [39]. ADF was determined gravimetrically as the residue remaining after acid detergent extraction [40]. For TP, samples were processed via nitric acid/hydrogen peroxide microwave digestion, and then TP was quantitatively determined by inductively coupled plasma atomic mission spectrometry [41], [42].

To assess overall meadow wetness, individual monitoring sites were categorized along a relative wetness scale with scores ranging from 0 to 6, as integers. In 2008, sites were ranked based on dominant plant community, extent and timing of surface flooding and saturation, and soil characteristics (mineral vs. organic dominated soils, depth of peat accumulation in organic soils, abundance of redox features in mineral soils). For example, relatively drier grass/forb-dominated sites on mineral soils represented a 0 rank, seasonally wet sites co-dominated by forb and *Carex* species common to moist sites (e.g., *Aster alpigenus* and *Eleocharis* species) represented a 3 rank, and continuously flooded sites dominated by wetland obligate *Carex* species represented a 6 rank. Site rankings were assigned at the end of the growing season (i.e., period of maximum water table draw down) so that rankings reflected relative differences between sites regardless of the type (wet, average, or dry) of rainfall year (i.e., ranks were on a fixed scale). Site rankings were averaged within each meadow to provide composite meadow-scale hydrologic rankings. For example, a meadow with a dominant wet *Carex* community and a subdominant drier grass/forb community would have three monitoring sites in the *Carex* community (3×6 rank) and two monitoring sites in the grass/forb community (2×0 rank), resulting in a mean score of 3.6, which is rounded to a “4” meadow rank assignment. Rankings were calibrated at sites within 10 additional meadows in the study allotments, which were equipped with ground water wells. Depth to free water was measured approximately every four weeks throughout the grazing season [43].

Meadow-scale toad occupancy surveys were conducted for all 24 meadows during the early tadpole periods (Fig. 1) in 2007 and 2008. Meadows were systematically searched for all toad life stages (egg masses, tadpoles, metamorphs, subadults, and adults) by three-member crews, with search times adjusted for individual meadow size and ease of search (e.g., more search time was allocated to meadows with high standing crop biomass). Searches were conducted during the early season (June–July), when tadpoles (i.e., the most easily detectable stage) were still abundant. Based on pilot studies, searches were conducted during mid-morning hours (0900–1100 hours) on cloudless days, which maximized detection potential. Each survey season, 5 of the 24 study meadows were completely resurveyed three times within a five day period to assess detection accuracy.

**Figure 3:**
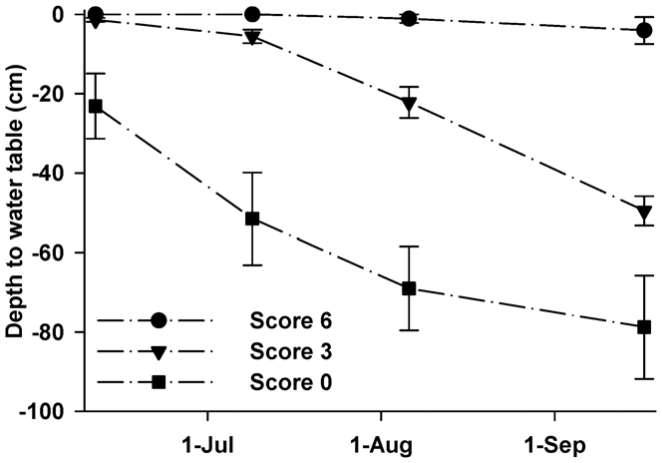
Water table dynamics. Mean depth to water table by meadow hydrology score for 10 meadows in the High Sierra Ranger District, Sierra National Forest, California, USA, during 2008. Hydrologic scale ranged from 0 to 6, with 0 representing drier sites and 6 representing the wettest sites. Water tables diverged over the summer: wet sites (score 6) experienced a mean seasonal drawdown of 4 cm while drier sites (score 0) experienced an mean seasonal drawdown of 79 cm. Vertical bars represent ±1 SE.

**Figure 4:**
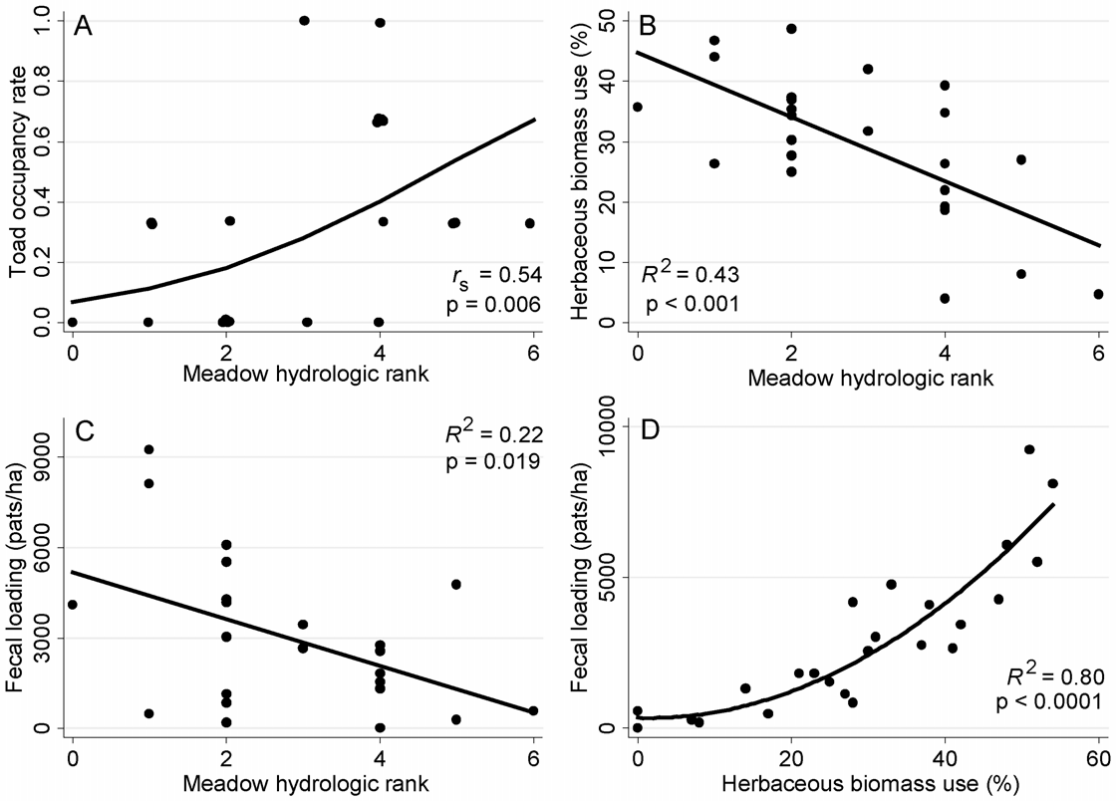
Toad and cattle meadow use. Toad occupancy and annual cattle utilization (percent herbaceous biomass use and fecal pat density) along a hydrologic gradient of meadows (n = 24) in the High Sierra Ranger District, Sierra National Forest, California, USA, during 2006 to 2008. Toad occupancy rate is calculated as proportion of surveys (three total; 2002/2003, 2007, and 2008) each meadow was occupied.

### Data analysis

#### Bivariate relationships

In order to provide proof of concept, supporting the construction of the general conceptual diagram for structural equation analyses (see next section), we examined the following bivariate relationships via multiple regression analyses (i.e., generalized linear and linear models): 1) meadow wetness and toad meadow occupancy rates, peak biomass production, herbaceous biomass use, and fecal loading; 2) fecal pat density and herbaceous biomass use, and 3) forage quality metrics and meadow wetness. Meadow wetness was measured as the composite meadow-scale hydrologic rankings (see Study design and data collection section). We also used generalized linear models to examine potential bivariate relationships between toad occupancy rates and cattle utilization (i.e., total herbaceous utilization), and to investigate the possibility of an interaction between cattle utilization and meadow wetness in predicting toad occupancy rates. Site rankings used to calculate the composite meadow-scale hydrologic ranks were normally distributed. For the bivariate analyses, toad meadow occupancy rates were calculated as the proportion of surveys (three total, including preliminary Forest survey and 2007 and 2008 surveys) toads were observed in each meadow. For meadow wetness relationships, peak biomass production and late season herbaceous biomass use (i.e., total use) were averaged over 2006 to 2008 for each meadow. Fecal loading was calculated as fecal pat density in 2008, and was related to 2008 herbaceous biomass use. Forage quality metrics were averaged for 2007 and 2008 for each meadow and related to mean (2007 and 2008) late season herbaceous biomass use.

All regression analyses were conducted in STATA [44]. Because toad occupancy rate is a proportional response variable, fractional logistic regression models [45] were used to fit toad occupancy rates by meadow wetness and cattle utilization (i.e., total herbaceous utilization). For these generalized linear models, normality of deviance residuals [46] and Spearman rank correlation for the model predicted values and observed values [47] were utilized to assess general goodness of fit. The remaining bivariate relationships were fit with linear and quadratic regression models. AIC and significance tests were used to select final models. Standard diagnostic analyses were utilized to check assumptions of linearity, normality, and constant variance. Box-Cox transformations were used to remedy any violations [44].

#### Bayesian structural equation analyses

After conducting exploratory bivariate analyses, we used SEM to examine expected pathways between meadow wetness, cattle utilization, and toad occupancy of meadows. SEM is a multivariate analysis technique combining path and factor analyses that permits evaluation of potential causal pathways of intercorrelated variables [31], [48]. The Bayesian approach offers greater flexibility than classical frequentist approaches to SEM. Unlike classical maximum likelihood estimates, Bayesian inferences do not rely on asymptotic normality, and so these estimators are more reliable for smaller samples or cases with other sources of non-normality [30], [49].

We began by constructing a conceptual SEM that incorporates the major known and hypothesized pathways of influence in the study system (Fig. 2). Within meadow ecosystems, it has been well established that spatio-temporal variation in depth to water tables exerts strong controls on plant community composition [22], [50]. Given this generally accepted relationship and the specific confirming results of above bivariate analyses, our conceptual SEM is based on the following: 1) via controls on community composition, meadow wetness influences plant community characteristics (i.e., productivity and forage quality), which are potentially correlated; 2) herbaceous biomass use by cattle is influenced by forage quality and productivity; 3) toad meadow occupancy is directly influenced by meadow wetness, which determines habitat suitability; and 4) toad meadow occupancy is directly influenced by cattle grazing (e.g., via impacts on physical and water quality attributes of toad habitat, trampling of individuals).

For SEM analysis, we used logistic regression to model the binary (i.e., present/absent) response variable for toad occupancy and linear regression to model all other normally distributed variables within a hierarchical (i.e., multi-level) framework. To account for non-independence of repeated measurements within meadows, random effects (i.e., intercepts) for meadows were included in the models, and to account for possible higher-level grouping and elevation differences (enrolled grazing allotments spanned an elevation gradient), meadow effects were nested within grazing allotments. To account for possible mean differences among years, random effects for year were also included [51], [52], [53].

**Figure 5:**
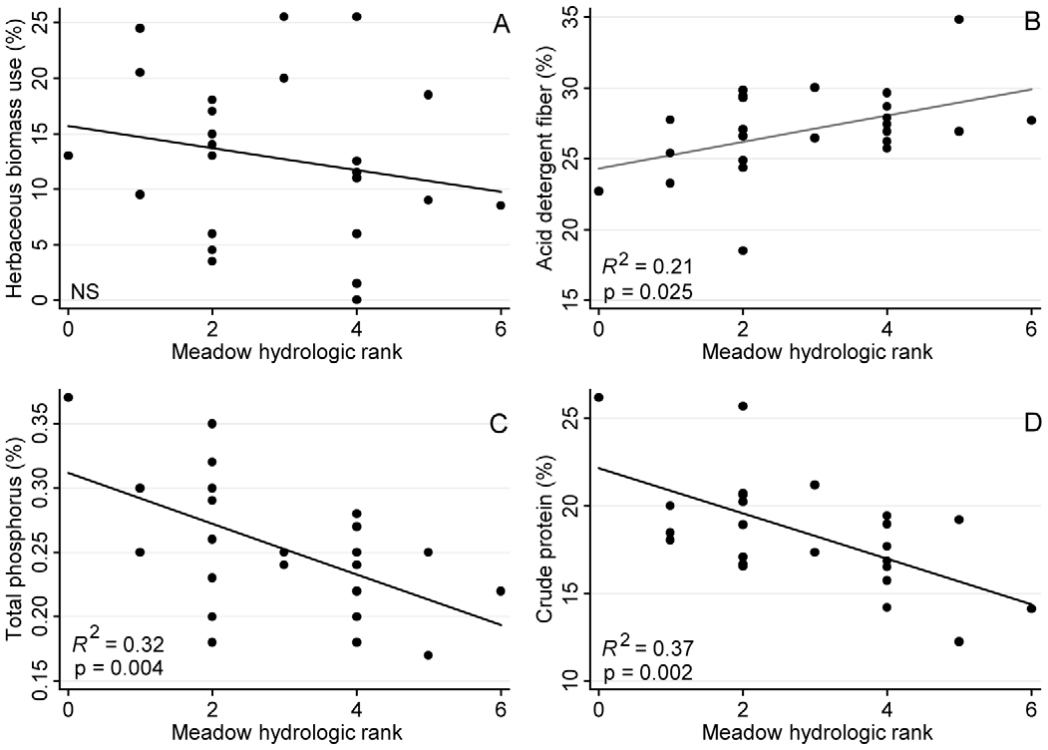
Early season bivariate analyses. Early season (July) meadow scale cattle use and forage quality along a hydrologic gradient of meadows (n = 24) in the High Sierra Ranger District, Sierra National Forest, California, USA. There was no significant trend in cattle use, as measured by mean early season herbaceous biomass use, across the meadow hydrologic gradient (panel A). Forage quality (crude protein, total phosphorus [TP], acid detergent fiber [ADF]; panels B–D) significantly declined with increasing meadow hydrologic rank (i.e., meadow wetness).

Bayesian SEM analysis was performed with OpenBUGS software, which uses Markov chain Monte Carlo (MCMC) simulation based on Gibbs sampling algorithm to fit the models [54]. For SEM, we analyzed herbaceous utilization, forage quality, biomass production, and meadow-scale toad occupancy data from 2007 and 2008 collection events, in addition to the one-time meadow hydrologic ranks. All continuous variables were standardized (mean = 0, standard deviation = 1) to aid model convergence and allow for direct comparisons of model coefficients. Model convergence was assessed utilizing trace plots with multiple chain sample values and a modified Gelman-Rubin statistic [55]. Model comparisons and goodness of fit were performed via the Deviance Information Criterion (DIC), a generalization of Akaike's Information Criterion (AIC) [56]. Statistical significance of individual model coefficients was examined via credible intervals (i.e., Bayesian equivalent of confidence intervals); coefficients were scored as significant when their 95% credible intervals excluded zero. To evaluate predictive capacity for toad occupancy and provide an additional measure of model fit, we cross-validated each model [57]. Each data point was held out and predicted by the model developed from the remaining *n-1* data points via the R statistical package rjags [58], [59]. Prediction errors for toad occupancy were assessed via receiver operating characteristic (ROC) curves, which are widely used to assess performance of presence/absence models in habitat conservation research [60], [61]. The accuracy of the predictors is measured by the area under the ROC curve (AUC), which ranges from 0.5 (no better than random) to 1 (perfect). Although no standard classification rules exist, AUC values greater than 0.70 are generally considered good, and values greater than 0.90 are considered excellent [62].

## Results

### Conditions during study period

During the study period, annual precipitation was 146.5 cm in 2006 (127% of average), 68 cm in 2007 (59% of average), and 84.4 cm in 2008 (73% of average). For the overall study period (2006 to 2008), study meadows represented a mean annual cattle herbaceous vegetation use gradient from 4 to 49%, and an annual biomass production gradient from 1000 to 3200 kg•ha^−1^. Mean forage production for early, mid, and late seasons was 723 kg•ha^−1^ (+/−39 SE), 1660 kg•ha^−1^ (+/−127 SE), and 1774 kg•ha^−1^ (+/−98 SE), respectively. Meadow wetness scores sufficiently reflected the seasonal water table variation between meadow sites with “drier” (score 0), “moderately wet” (score 3), and “wettest” (score 6) hydrologic rankings in meadows equipped with ground water wells (Fig. 3). Water table depths diverged over a four month period (2008 year), with hydric sites remaining flooded throughout the season and drier sites experiencing a seasonal drawdown of approximately 55 cm. Repeated searches of meadows in both survey years resulted in zero false negatives, confirming that single mid-morning searches were sufficient in accurately detecting species presence. For each survey, meadows were designated as toad occupied if evidence of breeding was found (i.e., presence of egg masses, tadpoles, and/or recent metamorphs).

### Bivariate relationships

Toad meadow occupancy rates (out of 3 total surveys) were positively correlated with meadow wetness (fractional logistic model p = 0.006, Spearman rank correlation [r_s_] of predicted vs. observed values = 0.54; Fig. 4, panel A), while mean cattle utilization was negatively correlated with meadow wetness (herbaceous use: *R* ^2^ = 0.43, p = 0.0005; fecal pat density: *R* ^2^ = 0.22, p = 0.019; Fig. 4, panels B and C). Mean peak biomass production was also negatively correlated with meadow wetness (*R* ^2^ = 0.21, p = 0.026). In the fractional logistic regression model for toad occupancy rates, neither cattle utilization nor the interaction of cattle utilization by meadow wetness were significant (p>0.1). There was a strong, significant relationship (*R* ^2^ = 0.80, p<0.0001) between the 2008 late season use and fecal loading metrics (Fig. 4, panel D).

**Figure 6:**
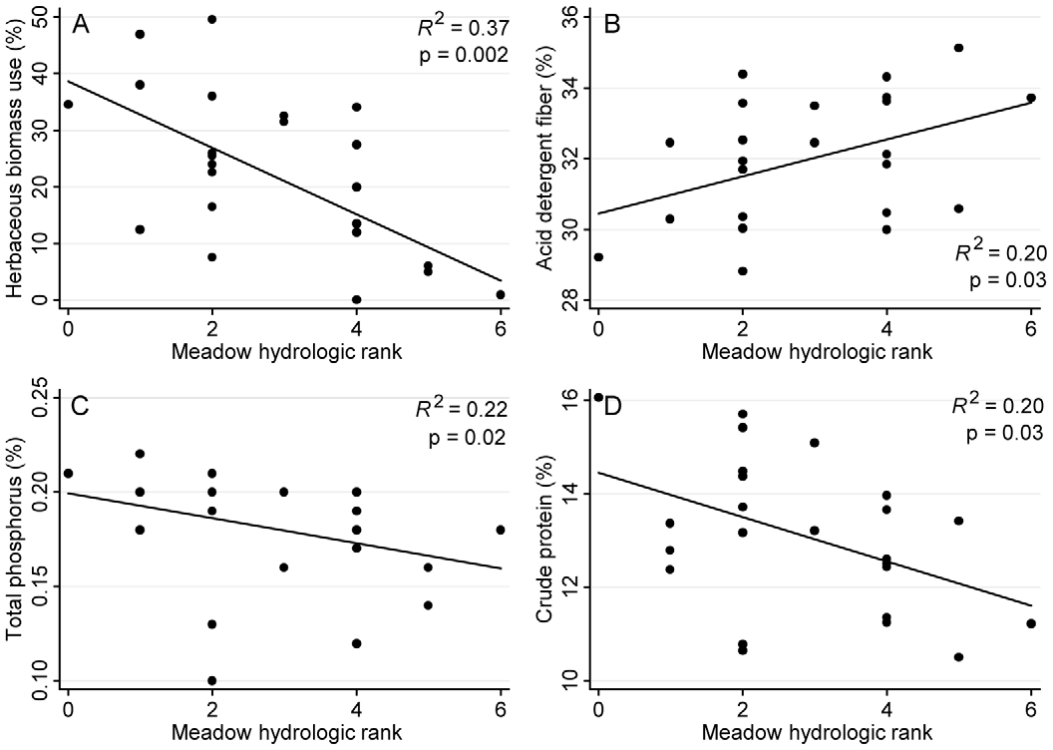
Mid season bivariate analyses. Mid season (August) meadow scale cattle use and forage quality along a hydrologic gradient of meadows (n = 24) in the High Sierra Ranger District, Sierra National Forest, California, USA. Cattle use, as measured by mean early season herbaceous biomass use (panel A), and mean forage quality (crude protein, total phosphorus [TP], acid detergent fiber [ADF; greater ADF values indicate lower digestibility]; panels B–D) significantly declined with increasing meadow hydrologic rank (i.e., meadow wetness). Late season (September) data exhibited similar trends.

Analyses of the 2007 and 2008 cattle use and forage quality data revealed few differential relationships across the three grazing seasons. There was no significant relationship between herbaceous biomass use and meadow wetness during the early season (Fig. 5, panel A); however, there were significant negative relationships between meadow wetness and herbaceous biomass use for both mid and late seasons (Fig. 6, panel A; late season data not shown). For all grazing seasons, forage quality metrics (ADF, TP, CP) were negatively correlated with meadow wetness (Figs. 5 and 6; late season data not shown).

### Bayesian structural equation modeling

**Figure 7:**
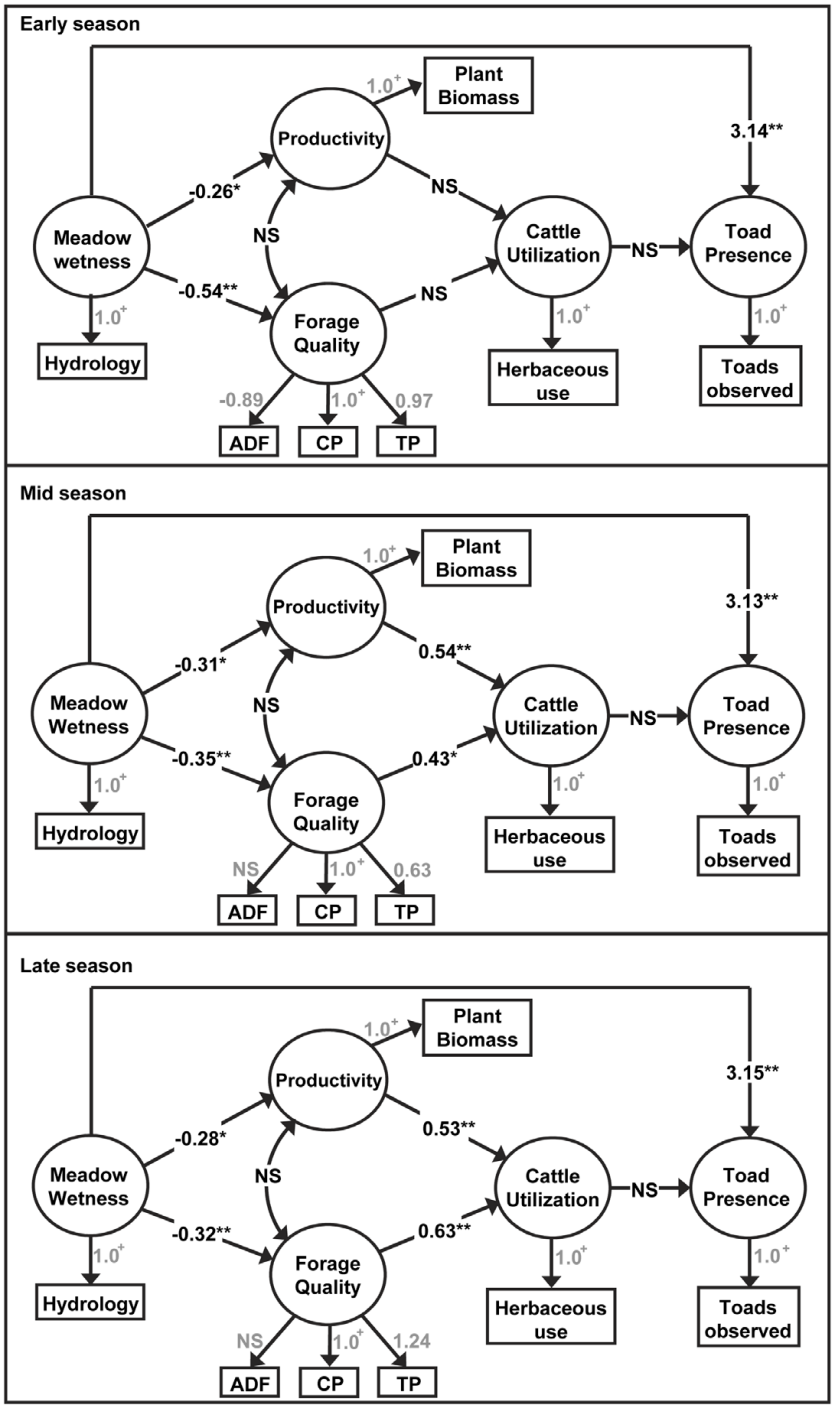
Bayesian structural equation model analyses. Results of Bayesian structural equation modeling for early, mid, and late season cattle use and forage data for the High Sierra Ranger District, Sierra National Forest, California, USA. All receiver operating characteristic (ROC) area under curve (AUC) values, which measured the accuracy of the predictors for toad occupancy, were ≥ 0.83. All models suggest toad presence responds to variation in meadow wetness, rather than cattle utilization levels. + = fixed values, ** = 95% Bayesian credible interval, * = 90% Bayesian credible interval, NS = Not significant.

Bayesian SEM results for all grazing seasons suggest toad presence strongly responded to variation in meadow wetness, but did not respond to variation in cattle utilization (Fig. 7). Direct effects of cattle use on toad meadow occupancy were not significant (utilizing 90% Bayesian credible intervals) for any season. For all grazing seasons, meadow wetness significantly influenced forage quality and productivity, which were not significantly correlated (Fig. 7). Cross validations for toad occupancy predictions produced reasonably good ROC AUC values for all grazing seasons: early, mid, and late season model ROC AUC values were 0.830, 0.832, and 0.832, respectively. Along with the DIC indicators used to compare relative fit among models, these metrics indicate reasonable model fit.

Across the grazing seasons, cattle utilization responded differentially to meadow forage quality and productivity. Early season cattle utilization did not significantly respond to any of the measured forage quality or productivity indicators (i.e., plant biomass production, ADF, TP, or CP). During the early season, forage quality fully met the general nutrient requirements of CP and TP (approximately 8% and 0.20% respectively) for lactating beef cattle [63], and forage production was limited across meadows early in the herbaceous growing season. Productivity exhibited a greater relative effect (0.54 vs. 0.43) on cattle utilization during the mid grazing season, while forage quality had a greater relative effect (0.53 vs. 0.63) during the late grazing season. Comparing the relative importance of CP and TP as indicators of forage quality, CP was relatively more important (1.0 vs. 0.63) during the mid grazing season, while TP became relatively more important (1.24 vs. 1.0) during the late grazing season. Mean TP fell far below general nutrient requirements (mean = 0.136%, range = 0.076 to 0.174) during the late season. ADF was a significant indicator of forage quality only in the early season analysis. ADF values were generally low throughout the entire grazing season, ranging from 15% to 39%.

## Discussion

Our study results suggest Yosemite toads and cattle largely select for divergent meadow types based on habitat and forage values, respectively (Figs. 4 and 7). Yosemite toads depend on meadows for vital breeding and rearing habitat, which is more abundant in wetter meadows. Wetter meadows provide greater habitat value for amphibians, which often exhibit metapopulation-like dynamics [64], [65], and potentially serve as source sites for overall population growth. Past habitat use surveys have shown that, in absence of cattle grazing, more than 50% of Yosemite toad subadults and adults are found in wet meadow bottoms, which provide persistent breeding and rearing pools [66]. These hydric zones are less likely to experience early season dry down (i.e., before tadpoles complete metamorphosis) than sites positioned higher in the meadow catena. Therefore, at the allotment scale, wetter meadows provide higher quality breeding and rearing habitat than relatively drier meadows, which provide more marginal habitat.

For cattle, wetter meadows provided relatively lower forage value for the majority of the grazing season. In the mid and late grazing seasons, cattle targeted relatively drier meadows, which supported more productive and nutritious plant communities, meeting general cattle nutrient requirements. As the grazing season progressed, forage quality became an increasingly important driver of cattle meadow selection as nutrient content declined with plant maturity (Fig. 7). However, during the early grazing season, forage quality was generally high and production was limiting across all meadows, resulting in relatively uniform grazing levels across all meadows. Despite these apparently uniform early season grazing levels (Fig. 5), cattle utilization did not significantly impact toad occupancy (Fig. 7). In this extensively grazed system, grazing intensities were light to moderate, with mean end of season use ranging from 4% to 49%. Fecal pat density, which serves as an indicator of longer term use patterns (i.e., given low environmental decomposition rates), was highly and significantly correlated with end of season cattle utilization (Fig. 4, panel D), indicating that use during the study period was indicative of cattle use over the past 5 to 10 years. Therefore, there are potentially two co-occurring mechanisms driving the overall lack of direct connection between cattle grazing and toad occupancy in this system: 1) for the majority of the grazing season, the two species mostly occupy differing zones along the moisture gradient, resulting in physical partitioning of the meadow habitat and minimizing any potential direct or indirect negative impacts; 2) when there is habitat use overlap (e.g., during the early part of the grazing season) grazing levels are low to moderate, resulting in no detectable impacts on toad occupancy.

Previous studies have reported negative associations between amphibian abundance and cattle grazing, indicating that amphibian species avoid or are excluded from livestock use areas. Following from this work, many have suggested cattle grazing activities reduce habitat value, citing potential mechanisms such as vegetation removal and degraded water quality [9], [11], [12], [13], [14], [29]. However, much of the existing cattle-amphibian work does not explicitly quantify grazing intensity, or has focused on grazed and ungrazed conditions in intensively grazed agro-ecosystems. Such comparisons generally offer limited application to extensive grazing systems, which commonly experience a continuum of grazing pressure. In a concurrent study within the same grazing allotments, our research group found no evidence that existing USFS grazing management impaired amphibian habitat conditions (i.e., water quality and cover) [67]. Other cattle-amphibian interaction studies from extensively grazed systems have demonstrated results similar to ours. In northeastern Oregon, an observational survey found no significant effects of extensive, moderate grazing on Columbia spotted frog (*Rana luteiventris*) reproduction [8]. Additionally, manipulative grazing experiments in the same region found no significant differences between grazed and ungrazed ponds in Columbia spotted frog egg mass counts, larval survival, or size at metamorphosis [68]. They also reported that nutrient levels were low or at minimum detection limits for all grazing and control treatments [68].

Our study clearly illustrates the importance of meadow wetness, and therefore hydrologic function, in determining toad occupancy. Loss of this critical wet meadow habitat will have direct negative impacts on Yosemite toad populations and other sensitive or threatened amphibian species. Some factors likely to negatively impact meadow hydrology and habitat availability include climate change, forest successional dynamics under altered natural fire regimes, and improper grazing management. Research at Yellowstone National Park has shown changes in climate (i.e., increased frequency and severity of drought, decreasing snowpack, and earlier runoff) and resultant wetland desiccation over the past 60 years were significantly correlated with declines in amphibian populations and species richness [69]. Climate models for the Sierra Nevada region suggest mountain meadows may be further threatened by predicted changes in future water yields, which will potentially result in overall longer periods of low flow conditions [70]. Shifts in both climate and fire regimes also alter forest successional dynamics, resulting in landscape-scale changes in vegetation cover [71], [72], which can potentially influence watershed-scale runoff and water yield [73]. Lastly, improper grazing management (e.g., heavy grazing, above levels reported in this study and above levels allowable by USFS regulations) can destabilize riparian areas and potentially lead to down-cutting and wetland desiccation via reduction in plant rooting mass and functional shifts in plant community composition [74], [75], [76]. Therefore, future habitat conservation practices for amphibian species of concern should focus on potentially critical factors directly impacting meadow hydrologic conditions, including climate change and land use activities such as heavy grazing, logging, and road construction.
